# Impact of a Graphene Oxide Reducing Agent on a Semi-Permeable Graphene/Reduced Graphene Oxide Forward Osmosis Membrane Filtration Efficiency

**DOI:** 10.3390/membranes11090679

**Published:** 2021-08-31

**Authors:** Grzegorz Romaniak, Konrad Dybowski, Anna Jędrzejczak, Anna Sobczyk-Guzenda, Bartłomiej Januszewicz, Witold Szymański, Paulina Kowalczyk, Tomasz Kaźmierczak, Jan Siniarski, Piotr Kula

**Affiliations:** 1 Faculty of Mechanical Engineering, Institute of Materials Science and Engineering, Lodz University of Technology, 1/15 Stefanowskiego St., 90-924 Lodz, Poland; konrad.dybowski@p.lodz.pl (K.D.); anna.jedrzejczak@p.lodz.pl (A.J.); anna.sobczyk-guzenda@p.lodz.pl (A.S.-G.); bartlomiej.januszewicz@p.lodz.pl (B.J.); witold.szymanski@p.lodz.pl (W.S.); paulina.kowalczyk@edu.p.lodz.pl (P.K.); piotr.kula@p.lodz.pl (P.K.); 2 Amii Sp. z o.o., 22 Techniczna St., 92-518 Lodz, Poland; tech@amii.pl (T.K.); jan@siniarski.pl (J.S.)

**Keywords:** graphene, graphene oxide, graphene membrane, desalination, forward osmosis

## Abstract

Graphene has been considered as a material that may overcome the limitations of polymer semi-permeable membranes in water treatment technology. However, monolayer graphene still suffers from defects that cause leakage. Here, we report a method of sealing defects in graphene transferred onto porous polymer substrate via reduced graphene oxide (rGO). The influence of various reducing agents (e.g., vitamin C, hydrazine) on the properties of rGO was investigated by SEM, Raman, FTIR, and XRD. Subsequently, membranes based on graphene/reduced graphene oxide were tested in a forward osmosis system using sodium chloride (NaCl). The effect of the effectiveness of the reduction of graphene oxide, the type and number of attached groups, the change in the distance between the rGO flakes, and the structure of this material were examined in terms of filtration efficiency. As a result, semi-permeable centimetre-scale membranes with ion blocking efficiency of up to 90% and water flux of 20 mL h^−1^ m^−2^ bar^−1^ were proposed.

## 1. Introduction

The rapid growth of the human population, industrialisation, and climate change have a great impact on decreasing water supplies. Along with the continuous release of pollutants into water such as microplastic, heavy metals, dyes, etc., these have caused over one billion people over the world to suffer from drinking water shortage [1]. For this reason, to maintain potable water, various techniques are used to remove impurities and microorganisms including sedimentation methods, electrodialysis (through ion exchange resins), distillation, and currently widely used are membrane technologies (microfiltration (MF), ultrafiltration (UF), nanofiltration (NF), reverse osmosis (RO), and forward osmosis (FO)) [2,3]. FO is a developing technology in which a semi-permeable membrane is placed between a feed solution and draw solution, and the separation process is driven by osmotic pressure across the membrane. The main advantages of FO are low energy consumption, a high rejection rate, and low membrane fouling [4]. FO can be used for desalination, wastewater treatment, as well as energy production. This process still packs membranes that will combine good strength properties and low thickness, while ensuring a high rejection rate and a high water flux [5].

The desire to develop a membrane with the smallest possible thickness, and thus, to obtain the lowest flow resistance, has prompted researchers around the world to work on the use of graphene and related materials for this purpose. Apart from the thickness of one atom (the thinnest possible active part of the membrane), the main advantages of graphene are its strength, flexibility, chemical, and thermal properties. Graphene, in its ideal form, cannot be used as a filtration membrane because it is impermeable even to atoms as small as helium [6], and, therefore, it is necessary to produce defects of certain sizes in it. For the water to be desalinated by the sieve effect, the size of the defects must be in the range from 0.3–0.7 nm [7]. Therefore, methods such as ion bombardment, electron beam interaction, and ion etching have been proposed so far to produce these types of defects [8,9,10].

Among the methods of synthesis of large-area graphene methods like CVD on liquid copper, CVD on solid copper, and HSMG^®^ can be listed. Quasi-monocrystalline HSMG^®^ graphene is produced by the metallurgical method on a substrate of liquid copper [11,12]. The method of producing this graphene is based on the use of the variable solubility of carbon in copper. The solubility of carbon in copper in the solid state is greater than its solubility in the liquid state. Graphene produced by this method is formed on liquid copper because of the separation of carbon supplied to the copper in a solid state. The nucleation of graphene seeds on the liquid enables their self-organization. The produced single graphene crystals can rotate on the liquid, creating a quasi-monocrystalline structure—the angle of disorientation of the grain boundaries of the formed graphene layer is less than 10 degrees.

The production efficiency of graphene membranes and their size are largely limited by the difficulty of obtaining large, continuous, and undefective graphene layers on mainly copper and nickel substrates. The synthesis of large-area graphene and its transfer to the support substrate determine the purity and quality of the produced membrane. Currently, the most used method of graphene transfer is a wet transfer, in which graphene is coated with a carrier polymer and then the graphene growth substrate is etched with appropriate reagents. A competitive method for wet transfer is electrochemical delamination, in which graphene is also coated with a carrier polymer, and then in the electrolyte, under the influence of the applied voltage, is separated from the substrate due to the hydrogen generated between the growth substrate and graphene [13,14]. It is a more effective method of transfer for graphene synthesized on a thick substrate (e.g., graphene HSMG^®^). Despite the development of the mentioned transfer methods, cracks and defects still appear at this stage and must be eliminated in the next steps of membrane production.

Currently, RO and FO membranes based on various graphene materials are being developed and tested. The active layers of such membranes are for example graphene monolayers, graphene multilayers, graphene monolayers sealed with polymer materials, layers of graphene oxide, and reduced graphene oxide applied by various methods that determine the final properties of the membrane. The membranes produced in this way are tested in the FO system, aimed at determining the speed of water transport and the reverse diffusion of ions. Osmotic stands with different salt concentrations (draw solution) and deionized water (feed solution) are used, or tests in FO systems that reflect the membrane work conditions more, where dextran draw solution and NaCl feed solution are used [7,15,16,17,18]. Additionally, composite membranes based on HSMG^®^ graphene sealed with reduced graphene oxide or nylon manufactured on polymer scaffoldings revealed semi-permeable properties in FO filtration mode. The clue of this solution was sub-nanometre pores in the graphene layer structure. The effectiveness of the graphene membrane depends on how it is sealed. Using graphene oxide for this purpose may be of key importance [19].

Graphene oxide contains graphite structure sheets decorated with oxygen-containing groups with a thickness of one or a few layers of carbon atoms. GO may be reduced via thermal reduction, chemical reduction, or multi-step reduction with the use of more than one method. As effective chemical reducers hydrazine, metal hydrates (e.g., NaBH_4_), ascorbic acid, hydroiodic acid, or hot strong alkaline solutions can be listed [20]. Besides reduction, hydrazine allows the creation of 3D structures based on cross-linked graphene or graphene oxide [21,22]. rGO may have different hydrophobicity, number of oxygen-containing groups, and better dispersion which may change the way of covering defects in graphene and, as a result, the performance of the membrane.

The aim of this work was to manufacture and investigate semi-permeable FO membranes based on HSMG^®^ large-area quasi-monocrystalline graphene and rGO. The graphene was transferred onto a polysulfone polymer nanofiltration membrane (PSU). Unlike other concepts, naturally occurring sub-nanometric defects in graphene were the active filter paths of the membrane and the key to the solution. The novelty is the use of reduced graphene oxide which allowed for sealing larger defects resulting from graphene growth and transfer steps. The analysis of graphene after its growth stage, as well as during transfer and after transfer to the polymer substrates (Raman, SEM, optical microscopy), was performed. Several reagents reducing graphene oxide were used and their influence on the morphology of the defect-sealing material was determined. Filtration tests were carried out in the FO system with the use of NaCl salt solution aiming to determine the dependence of semi-permeable properties on the graphene oxide reducing agent used.

## 2. Materials and Methods

### 2.1. Materials

Monolayer polycrystalline graphene for composite membranes preparation was synthesized via the metallurgical method (graphene HSMG^®^) according to the procedure described in detail in the patent (method of producing graphene from liquid metal, US 9 284 640, USA) and the article [19]. The synthesised graphene was qualitatively studied by Raman spectroscopy (inVia Renishaw Raman spectroscope) and TEM microscopy. Raman spectra obtained showed that graphene grown on the Cu-Ni substrate was a monolayer with a limited number of defects. A TEM Talos F200X from FEI was used to assess the nanostructure of the graphene layers produced. Based on these studies, nanostructural defects sized from 0.2 to 0.5 nm were identified and described in the previous work [19]. The importance of these defects in the structure of composite graphene membranes is also described there.

Porous polysulfone membranes were used as graphene supporting substrates (MicroPES^®^ 1F EL, made by Membrane GmbH, Radeberg, Germany). It was used to prepare composite graphene membranes in each test. Membrane thickness equaled 110 µm and was declared by the manufacturer as having water permeability of ≥10 mL min^−1^ cm^−2^ bar^−1^ at a temperature of 25 °C. We transferred graphene on a polysulfone membrane side with minor porosity where pore diameter was from 0.1 to 1.0 μm. The density of the pores was 1.05 × 10^6^ mm^−2^.

Poly(methyl methacrylate) (PMMA) with a molecular weight of 996,000 g/mol of the Aldrich Chemistry Company, Burlington, MA, United States, dissolved in chlorobenzene (Chempur, Piekary Śląskie, Poland), with a concentration of 0.10 mol dm^−3^ (9.2 g of PMMA-100 mL of chlorobenzene), was used for covering graphene during the transfer procedure.

The solvent used to dissolve and remove the PMMA foil from graphene during the transfer procedure was 2-propanol with a technical grade of 70% (Chempur, Poland).

Graphene oxide with 0.4% dispersion in water (4 mg/mL) was purchased from the Advanced Graphene Products Company, Nowy Kisielin, Poland.

For the reduction of graphene oxide, diluted commercial 80% hydrazine hydrate (POCh, Gliwice, Poland), pure ethylenediamine A.C.S (Chempur, Poland), 98.5 % sodium borohydride (NaBH_4_) A.C.S (Pol-Aura, Różnowo, Poland), L(+)-ascorbic acid (C_6_H_8_O_6_) A.C.S (Chempur, Poland), and green tea leaves commercially available in the local market, were used.

For the osmotic test, sodium chloride (NaCl) pure p.a. from Chempur, Poland, was used. For membrane assembly in the grip of side-by-side diffusion cell Polydimethylsiloxane (PDMS), SYLGARD™ 184 Silicone Elastomer (The Dow Chemical Company, Stade, Germany), was used.

### 2.2. Transfer of Graphene

The HSMG^®^ graphene transfer process onto a polysulfone membrane was conducted using the electrochemical delamination method [14]. The process begins with cutting samples of graphene on a Cu/Ni growth substrate with a size of 35 × 35 mm^2^. This was followed by drop/blade coating graphene with PMMA in chlorobenzene as a temporary carrier layer. The polymer was dried at a temperature of c.a. 40 °C for 30 min. Delamination of the HSMG^®^ graphene was carried out using a laboratory half-automatic station in 0.5 mol/L NaOH solution at a constant voltage of 4.5 V at a sample submersion angle of 35° and a submersion linear speed of 0.02 mm s^−1^. Then, graphene on PMMA foil was washed three times in deionized water. Graphene on polymer foil was placed on a wetted polysulfone membrane and roll pressed with a sponge roller and then annealed at 70 °C for 5 min. A gentle pressing was used to get rid of water and provide good attachment while avoiding damaging the graphene. Then it was placed in a chamber with boiling isopropanol to remove the PMMA-supporting layer. The graphene membrane was then washed twice in cold isopropanol to remove any polymer residues and air-dried.

After transfer on the target, substrate graphene was subjected to SEM analysis (HITACHI S 3000 N). In this way, we established that no new cracks and tears are introduced to graphene at any stage of the transfer method.

### 2.3. Reduced Graphene Oxide Preparation

Reduced graphene oxide (rGO), listed in Table 1, was used to cover the structural defects of HSMG^®^ graphene. It was prepared by the chemical reagent reduction of graphene oxide. rGO reduced with hydrazine without centrifugation of the reaction products was prepared via preparation of 1% solution of hydrazine in 50 mL of 0.4% GO in DI water suspension and left for 48 h to reduce at room temperature (RT).

The reduction of GO via hydrazine and ethylenediamine was conducted via preparation of 1% solution of hydrazine and ethylenediamine in 50 mL of 0.4% GO in DI water suspension, respectively. The solution was kept for 48 h at RT. Next, the rGO was centrifuged (9000 rpm, 10 min). After being centrifuged, the washing procedure was applied. The reaction mixture was poured out and 50 mL of DI water was added. In the next step, rGO sediment was extensively vortexed and mixed using an ultrasonic cleaner for 15 min. The washing procedure was repeated six times [21].

NaBH_4_ (0.4 g), as a reducing agent, was added to 50 mL of 0.4% GO in DI water suspension. To obtain rGO, the suspension-prepared mixture was kept with continuous stirring at room temperature for 12 h. Next, the suspension was washed according to the procedure described above [23].

In the next stage of the experiment process, the reduction was carried out using ascorbic acid (0.4 g) diluted in 50 mL of 0.4% GO deionised water suspension. The reaction mixture was kept for 48 h at RT with gentle stirring. After the reduction process, the rGO suspension was washed according to the procedure mentioned above [24].

The last reducing agent used to reduce graphene oxide was green tea leaf extract. 15 g of dried tea leaves were boiled with 200 mL of DI water at 80 °C for 1h in a 250 mL flask to obtain the tea leaf extract. Next, the prepared solution was filtered. The 50 mL GO solution and 50 mL tea leaf extract were heated to 90 °C and then mixed in a ratio of 1:1 (vol. ratio). The reduction of GO was carried out with continuous stirring at 90 °C for 12 h. After this time, the above-mentioned washing procedure was applied [25,26].

### 2.4. Reduced Graphene Oxide Characterization

Raman spectroscopy measurements were performed using an inVia Renishaw Raman spectroscope equipped with a 532 nm laser. The laser power during the measurements was reduced to the value of 0.3 mW to avoid sample damage, while laser exposition time was 10 s. A 50× objective was used for all measurements.

For the needs of spectral analysis, each peak was fitted to a single Lorentzian using the PeakFit software. The fits achieved an R^2^ greater than 0.98.

The infrared absorption of GO and rGO samples in the spectral range from 4000 to 500 cm^−1^ was measured by Fourier transform infrared spectroscopy (FTIR using a Thermo Scientific spectrometer, model iS50). Spectra were recorded at a resolution of 2 cm^−1^ using a highly sensitive MCT-B detector (Telluride Mercury Cadmium Telluride). Measurements were made in the reflection mode with the use of the Sequelle DRIFT reflection adapter, for the incidence of the radiation beam equal to 20 degrees. Spectra were collected from 256 scans.

The X-ray diffraction studies of graphene oxides were performed with the use of the PANALYTICAL Empyrean X-ray diffractometer (Panalytical, Almeo, Netherlands). The diffraction patterns were collected using a tube with a Cr anode-emitting characteristic radiation with a wavelength of λ = 2.291 Å, operating at a voltage of 30 kV and current of 55 mA. Due to the high roughness of the graphene oxide samples deposited on nickel substrates, on the primary radiation beam, an X-ray lens with a cross collimator was used. On the reflected beam path, a 0.18 deg parallel beam collimator, 0.04 rad Soller slits, and a proportional detector were installed. The tests were carried out in the angular range from 2θ = 10–60°, with a 0.05° step, and time per step equal to 5 s.

### 2.5. Membrane Preparation and Characterization

**Figure 1:**
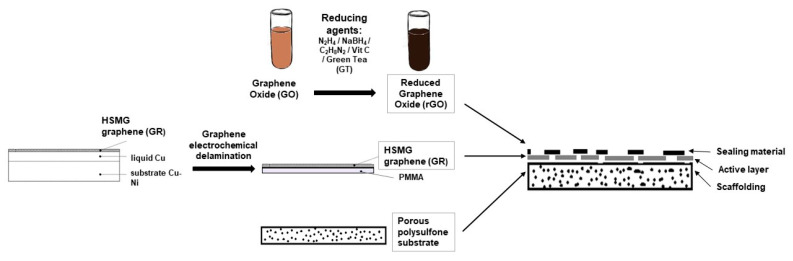
Graphene membrane preparation scheme.

The as-prepared rGO suspensions and unreduced GO suspension were dispersed in distilled water at a concentration of 0.1%, respectively. Then the solution was applied onto a polysulfone membrane covered with graphene HSMG^®^ in the amount of 30 µL per 1 cm^2^. After 5 min, the excess solution was washed off with a gentle stream of water. In the next step, another 10 µL/cm^2^ of rGO/water solution was applied on the graphene membrane and air-dried. Each time before use, the rGO solution was dispersed by means of ultrasound. Due to the hydrophobic nature of graphene, a water suspension of rGO tended to accumulate in graphene discontinuities (uncoated polysulfone area). In this way, graphene HSMG^®^ defects were sealed. The preparation of each membrane was performed in the same way as presented in Figure 1.

Once made, the graphene membranes were subjected to qualitative investigations. The tests were carried out after the transfer of HSMG^®^ graphene to the PS substrate and after sealing with reduced graphene oxide. The observation of the surface of the graphene membranes was performed using the HITACHI S 3000 N scanning electron microscope. The samples were placed in the chamber using a table pressed with a ring in such a way as to ensure good charge-discharge from the entire surface of the graphene composite area. The tests were performed at the intensity HV = 5 kV, in SE and AEE modes, which enabled the identification of defects and impurities in the structure. The contrast in AEE mode is the result of the differences in the electrical conductivity of the tested micro-areas.

### 2.6. Experimental Setup for Filtration Test

Ion separation effectiveness and water flow through membranes driven by osmotic pressure gradient were measured with a side-by-side diffusion cell with a 22 mm orifice. The volume of each chamber (DI water and salt solution) was 64 mL [19].

One of the cylinders was filled with 0.2 M NaCl as a draw solution and the second was filled with degassed, deionized, UV lamp-treated water as a feed solution.

The membranes were sealed in a grip with a semi-liquid polydimethylsiloxane polymer which was then polymerized. This provided membrane stabilization and good sealing while causing no damage to the working part of a membrane.

After being made, the graphene membranes were subjected to qualitative investigations. The tests were carried out after the transfer of HSMG^®^ graphene to the PS substrate and after sealing with reduced graphene oxide.

### 2.7. Water and Salt Transport Measurements

Each time after membrane assembly both cylinders with feed and draw solution were filled simultaneously to avoid membrane deflection. The tests were conducted at room temperature (20 °C) for 24 h. Each time the salt concentration was measured on each side of the membrane after the test. To evaluate water flux (J), the transport of water from the feed side to the draw side, driven by the osmotic gradient that resulted in a rise of water level in the draw side, was measured using a graduated glass tube during the test.

Each experiment was repeated twice by replacing the solution in each cylinder.

The osmotic pressure was calculated based on the van’t Hoff equation (π=νcRT), where ν is the number of ions in dissociated salt, *c* is the molar concentration of the solute, *R* is the gas constant, and *T* is the temperature in Kelvin. The calculated pressure was 9.7 bar.

The water flux through the membrane (J) (mL m^−2^ h^−1^ bar^−1^) was calculated based on the equation $J=\frac{V}{A\;t\;p}$, where *V* is the change of water volume in a container (mL), *A* is the membrane-active surface area (m^2^), *t* is time (h), and *p* is theoretical osmotic pressure owing to the concentration difference (bar). The ion’s flux (mol m^−2^ s^−1^) was calculated by dividing the change of salt concentration in the container with feed solution by the test time and the membrane surface. The degree of ion transport blocking efficiency was calculated relative to an uncoated polysulfone membrane (bare PSU), assuming 0% ion blocking efficiency is the same ion transport as bare PSU and 100% ion blocking is no ion transport.

## 3. Results and discussion

### 3.1. Reduced Graphene Oxide Characterization

#### 3.1.1. Raman Spectroscopy

**Figure 2:**
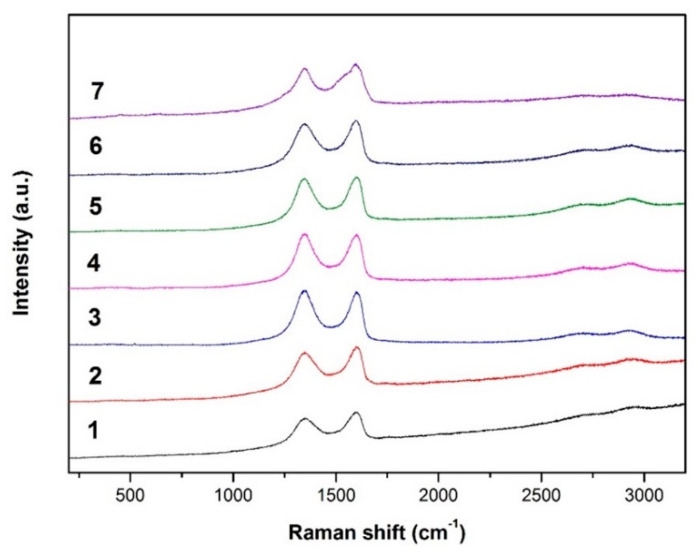
Changes in Raman spectra of the GO and rGO samples.

Raman spectra of the studied samples are placed in Figure 2 and represent a typical shape for both GO and rGO materials.

The spectra were deconvoluted using a Lorentz function in the range of the spectral region from 750 cm^−1^–2000 cm^−1^ in accordance with the method presented in the work [27]. In agreement with it, a Gapp peak located at ~1600 cm^−1^ was deconvoluted into two modes, G and D’. Then a difference of the positions of the Gapp and D’ was estimated. The results of the analysis are placed in Table 2.

In accordance with the work [27], difference in the D’ and G_app_ position (D’-G_app_) may be considered as a function of the C/O ratio:

GO = D’ _inf_-G _app_ < 0; C/O < 10

rGO = 0 < D’ _inf_-G _app_ < 25; 10 < C/O < 500

Graphene = D’ _inf_-G _app_ > 25; C/O > 500

In view of the above, in the case of sample No. 1 (graphene oxide, GO) the value of the difference (D’-G_app_) is equal to −0.52, at the same time fulfilling the condition D’ _inf_-G _app_ < 0, which is typical for GO.

The difference in the D’ and G_app_ positions for all the other samples equals more than 0, which points to the reduction of the oxygen concentration in relation to the carbon content (an increase of C/O ratio).

According to the Raman spectroscopy analysis, samples No. 2 and 3 (the hydrazine-reduced GO’s) show the highest value of (D’ _inf_-G _app_) amounting in both cases, ~13 (13.30 and 13.46, respectively). The value placed in this range is characteristic of the reduced graphene (rGO). The higher D’ and G_app_ difference, the more reduced the GO. Therefore, based on the obtained results it can be stated that the most effective reduction has been achieved by the reaction of GO with hydrazine, which is also confirmed by the FTIR and XRD analysis.

The other samples showing a high level of reduction are 5 (GO reduced with sodium borohydride) and 4 (GO reduced with L(+)-ascorbic acid). The difference between D’-G_app_ peak positions in these cases equals 7.26 and 5.01, respectively.

In Raman measurements, the least effective reduction of GO was obtained for samples No. 6 and 7, where (D’-G_app_) value was equal to 1.79 and 0.5, respectively, which means that both ethylenediamine and green tea turned out to be the least efficient reducing agents for graphene oxide in these experiments. However, in the case of the reactions with green tea the lowest level of the reduction of GO was obtained, which is also confirmed by the XRD and FTIR analysis.

#### 3.1.2. FTIR

**Figure 3:**
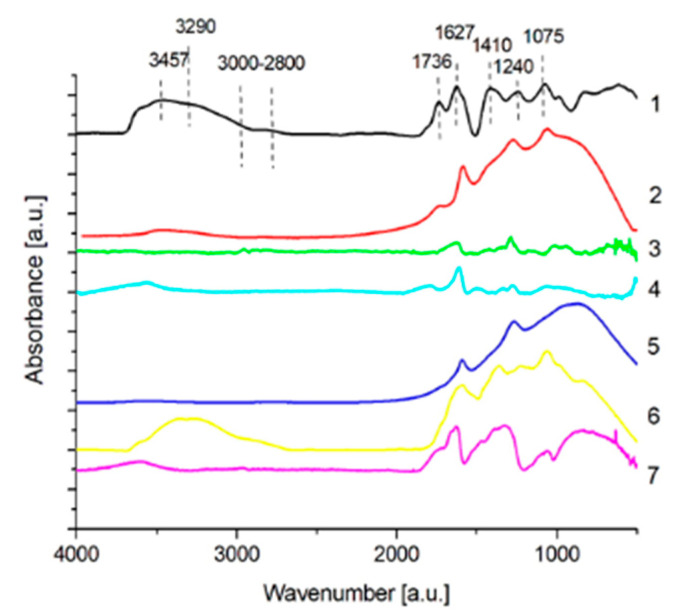
FTIR spectra of unmodified GO and GO reduced with various reducing agents.

Figure 3 presents the FTIR spectra of graphene oxide (GO), both unmodified and reduced with various reducers such as N_2_H_4_, ascorbic acid, NaBH_4_, GT, and C_2_H_8_N_2_.

The spectrum of unmodified GO shown in Figure 3 comprises all the bands characteristic for the chemical structure of that material. First of all, at the wavenumber of 1736 cm^−1^, the presence of an absorption band originating from stretching vibrations of C=O bonds in the carboxyl group has been confirmed. Such a group may be attached to the graphene structure both along the edge of its sheet and directly in its base plane. In addition, the presence of the carboxyl group is confirmed by the maximum at 3457 cm^−1^, corresponding to the stretching vibrations of the hydroxyl bond. Another absorption band at 3290 cm^−1^ reveals a presence of a fairly large number of O-H groups on the surface of GO, derived from water of the reaction environment and linked together in a network by hydrogen bonds. Finally, as far as deformation vibrations of the C-O-H bond system in the carboxyl group are concerned, they give rise to the maximum at 1410 cm^−1^. Another bond system characteristic for GO is the epoxy group (C-O-C) identified by the adsorption bands at 1240 cm^−1^ and 1075 cm^−1^ derived from the stretching vibrations of the C-O bonds. In addition, at 1627 cm^−1^, there is a typical maximum for stretching vibrations of C=C bonds taking place in the hexagonal ring of graphene oxide [28,29,30]. In the wavenumber range between 3000 and 2800 cm^−1^, a wide band also appears, originating from both symmetric and asymmetric stretching vibrations of C-H bonds in aliphatic hydrocarbon chains attached to the GO ring structure [30]. Such moieties constitute an impurity of this material, most likely formed during the synthesis of this material.

When it comes to GO reduction, hydrazine, sodium borohydride, and ascorbic acid turn out to be the best reducing agents and this can be observed in the IR absorption spectra. First of all, following reduction the band originating from the C=C vibrations shifts away from the position dedicated to GO. In hydrazine reduced material, the bands originating from hydroxyl and carboxyl groups disappear, while groups typical for C=N connections in the range of 1690–1640 cm^−1^ (especially visible for reduction with hydrazine with centrifuged reaction products) and at 1280 cm^−1^, which correspond to the stretching vibrations of C-N bonds, emerge [30,31]. In addition, in the spectra of both centrifuged and non-centrifuged hydrazine-reduced material, a peak at 1580 cm^−1^, corresponding to the stretching vibration of N-H bonds and characteristic for the NH_2_ group, appear. Additionally, in the spectrum of graphene reduced with non-centrifuged hydrazine, a maximum at 1530 cm^−1^, corresponding to the C-NH_2_ stretching vibrations, emerges along with a wide and distinct band in the range from 3500–3300 cm^−1^ related to the stretching vibrations of N-H bonds. This means that hydrazine centrifugation is necessary to clean the surface of the reduced GO to get rid of the toxic residues of the reducer used. Nitrogen-containing compounds allow not only for the reduction of GO but also the formation of bridges between carbon atoms in different rGO flakes [21,22]. On the other hand, following reduction with vitamin C, a wide band belonging to the vibrations of hydroxyl groups (constituting a component of the -COOH group) disappears, while a weaker maximum appears, which shifts towards the range corresponding to free hydroxyl groups. As in the case of the reducing effect of hydrazine, the peak from the -COOH group and the majority of the peaks belonging to the C-O-C and C-O bonds also vanish. It is believed that epoxy groups in GO can be easily approached by nucleophilic reagents, resulting in a nucleophilic substitution reaction leading to the opening of epoxy rings. Easily detachable (-OH) groups appear in the opening epoxy rings and eventually the structure “dehydrates”, leaving a sheet of reduced graphene oxide. It is believed that for the removal of one -OH group, ascorbic acid donates one proton to the -OH group [32].

Another effective reducing agent is NaBH_4_. After reduction with this substance, no maxima assigned to -OH and -COOH groups are observed but, instead, an intense band at 1270 cm^−1^ originating from epoxy groups is present. In addition, a new peak emerges at 1592 cm^−1^, related to the presence of COO^-^ anions, which is a consequence of the presence of the sodium cation in the reducer used and the formation of the carboxylic anion [31].

Only in the cases of GO treated with GT and C_2_H_8_N_2_, the C=C peak is still at the constant 1627 cm^−1^ position. In the spectrum of GO reduced with green tea, apart from signals corresponding to carboxyl groups, epoxy, and other C-O connections characteristic for GO, absorption bands typical for this reducing agent, appear. Moreover, the presence of a maximum at 1655 cm^−1^ assigned to the stretching vibrations of the C=O bond in first-order amides, as well as that at 1327 cm^−1^, due to the stretching vibrations of aromatic amines of I and II order [30,31], is revealed. As far as the absorption bands at 1084 cm^−1^ and 1056 cm^−1^ are concerned, they originate from stretching and deformation vibrations, respectively, of C-O bonds in the C-OH group of polysaccharides [33]. Finally, in the case of ethylenediamine-reduced GO, the presence of all the bands characteristic for the GO spectrum has been identified. In addition, maxima corresponding to the vibrations of the N-H bond in both -NH- and -NH_2_ amine groups are recorded in the spectral range from 3300–3150 cm^−1^ (stretching vibrations) and at 1580 cm^−1^ (deformation vibrations of N-H bonds in the -NH_2_ groups), and those originating from C-N bond stretching vibrations are observed at 1220 cm^−1^ [30,31]. Compared to other modifiers, the number of C-H bonds belonging to -CH_2_- and -CH_3_ groups is also higher in ethylenediamine-reduced GO.

#### 3.1.3. XRD

**Figure 4:**
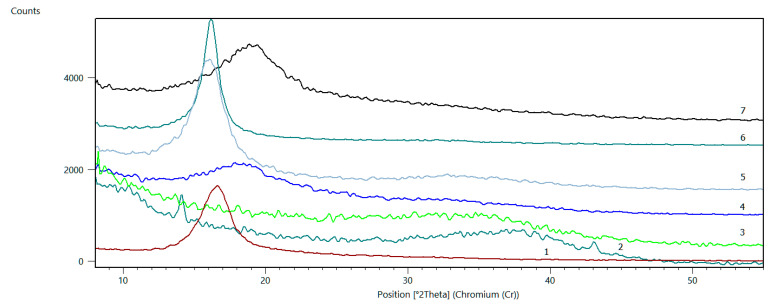
Diffraction patterns of GO and rGO.

Figure 4 shows the diffraction patterns of all the investigated samples, described in Table 2. These spectra show the characteristic diffraction peak positions for GO and rGO, obtained depending on the reductant used.

The reflection mode geometry X-ray diffraction pattern for a neat GO has a strong (001) diffraction peak indicating the preferred orientation of graphene oxide basal planes parallel to the sample plane. The two-theta position of the (001) GO diffraction peak can show a range from ~7–12 2theta (Cu Kα radiation) that corresponds to ~10–18 2theta for Cr Kα radiation, depending on the amount of residual water intercalated between basal planes in a GO film [34]. Interplanar distance for GO in Sample No. 1 is equal to 7.972 Å, a value in the range reported in the literature [35,36]. Based on the (001) peak width, the crystallite size was determined using the Scherrer equation and was found to be 25.7 Å in the [00l]-direction.

An average number of layers per flake of GO and r-GO is calculated using the flake size and d-spacing parameters obtained from XRD analysis.

$$n=\left(\frac{D}{d_{\left(001\right)}}+1\right)$$

Here, *n* is the number of layers, *D* is the crystallite size, and *d*(001) is the interplanar distance.

The results of the explained estimations above for all samples are summarized in Table 3.

In sample No. 2 intense reduction of GO can be observed, but calculations of d, D, and n values also indicate a very intense conglomeration of remaining GO flakes and likely the exchange of pre-existing H-O bonds between flakes with reducing agent species. This effect is likely also related to the lack of centrifugation, the product of the hydrazine reduction reaction, and the formation of C-N, C=N bonds.

In the case of sample No. 4, reducing agents caused the removal of H-O species from between GO flakes and the partial transformation into rGO, which is indicated by decreasing the d for GO and the appearance of the rGO peak.

For sample No. 5, the reducing agent, however, caused a partial reduction of GO to rGO, but at the same time, the increment in d’s is observed and in crystallite dimensions.

For sample No. 6, there is a shift towards lower 2Th values only. No rGO peak was observed which can indicate the applied reducing agent species added to existing H-O ones. In sample No. 7, the decrease of d interplanar spacing of GO is observed, however, no rGO peak appeared. The calculation of the “n” value suggests that the applied agent only caused the division of GO flakes without any reducing effect.

In the examined group of samples, only sample No. 3 shows the complete reduction of GO without any additional effects.

In all cases created, rGO is characterised by very close d, D, and n values.

### 3.2. Composite Graphene/Reduced Graphene Oxide Membranes Characterisation

#### 3.2.1. Effectiveness of Sealing Membranes with rGO–SEM Characterisation

The membranes were manufactured and labeled as described in Table 1.

Table 4 (sample No. 8) shows the SEM, SE, and AEE images of the surface of the polysulfone membrane with an HSMG graphene monolayer without GO/rGO. There are visible defects of the graphene monolayer with sizes ranging from a few to several hundred micrometers, formed in the HSMG^®^ polycrystalline graphene at the stage of synthesis and/or transfer to a polysulfone substrate. On membrane 1, defects in the graphene layer were masked with graphene oxide. Large GO conglomerates covering the surface of HSMG^®^ graphene are visible here. Membranes 2 and 3 were sealed with reduced graphene oxide as a result of the use of hydrazine. There is an even coverage of the rGO graphene surface, with no major conglomerates. As the AEE images show, there are no non-conductive sites, which proves a very good continuity of graphene layers supplemented with rGO. On membranes 4, 6, and 7 the coating is not as uniform as in the other cases (except membrane 1), some of the sealing material tended to accumulate in larger aggregates on the surface of the membrane, thereby forming large conglomerates GO/rGO. The surface of diaphragm No. 5 is also characterised by homogeneous coverage GO/rGO as for diaphragms 2 and 3. The wrinkles characteristic of graphene oxide/reduced graphene oxide can be seen here. The entire surface of HSMG^®^ graphene is covered with such a wrinkled coating. Furthermore, all images of membranes with transferred graphene show reflections of the boundaries of copper grains from the growth substrate, however, this does not affect the continuity of graphene in these areas.

#### 3.2.2. Forward Osmosis Performance of Membranes

**Figure 5:**
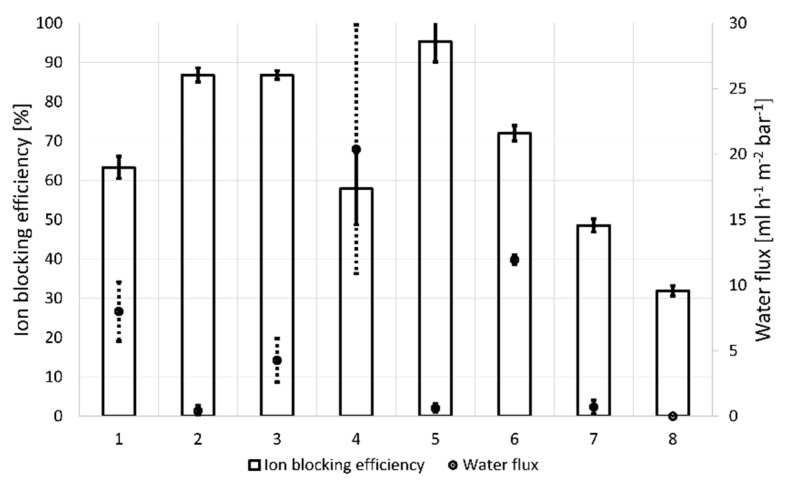
Forward osmosis test result, water flux, and ions blocking efficiency.

In the presented results of osmotic tests for the as-prepared composite graphene membranes, the effectiveness of blocking the ion transport ranges from 30–95%. For the membrane with unsealed graphene (No. 8), no semi-permeable properties were noted, it was characterized by high ion transport and no water flux caused by osmotic pressure (Figure 5).

A reference to other options for the composite membrane preparation is also the membrane sealed with non-reduced graphene oxide. A water flux achieved for it is over 8 mL h^−1^ m^−2^ bar ^−1^ and an ion blocking efficiency of approximately 60%.

The reduction of GO with hydrazine (sample No. 2) and its use as a material for sealing defects in graphene increased the ion blocking efficiency for the membrane prepared in this way up to 87%, however, a water flow close to zero was noted.

For membrane 3, in which a completely hydrazine-reduced graphene oxide was used as the sealing material, the same ion blocking efficiency as in membrane 2 was achieved, but with a significantly higher water flow. Here, in relation to membrane 1, ion blocking was higher at lower water flow rates. This phenomenon is associated with a reduced interplanar distance in the rGO flakes, a lower number of oxygen groups in reduced graphene oxide flakes, and, thus, the more accurate sealing of defects. In this case, the blocking of the ion flow and water molecules is also greatly influenced by the uniform coverage of the membrane surface by rGO and the low tendency to form conglomerates. On the other hand, the increase in water flow in membrane 3 with respect to membrane 2 with the same degree of ion blocking may be due to the greater presence of C=N and C-N bonds in membrane 3. The blocking of water flow through membrane 2 may also be influenced by non-centrifuged reduction reaction products.

The most intense water flow was achieved for the membrane with vitamin C-reduced graphene oxide. In this case, the effective removal of OH and COOH groups, the reduction of the distance between the planes of non-reduced graphene oxide, and the small distances between the planes of reduced graphene oxide translated into such a sealing of large defects in graphene that the highest water transport was obtained under the influence of osmotic pressure. The ability to selectively coat the surface of graphene HSMG^®^ also contributes to this high water flow. However, the resulting large rGO conglomerates, and the tendency to form clusters on the surface, do not sufficiently cover the defects in HSMG^®^ graphene. Therefore, the migration of ions through this type of membrane is high and the repeatability of the results is worse.

Sealing graphene with ethylenediamine-reduced GO resulted in a water flow of 11.5 mL h^−1^ m^−2^ bar^−1^ with an ion blocking efficiency of 70%. This is due to the very low degree of reduction of GO and the presence of nitrogen bonds, which further increased the interplanar distances, which was demonstrated in FTIR and XRD results. In this case also, conglomerate formation and GO aggregation on the HSMG^®^ graphene surface did not sufficiently cover the defects, but selective coverage resulted in high water flow.

Green tea turned out to be an ineffective reagent, which not only had almost no effect on the reduction of graphene oxide but also the transport of water and ions was close to the one obtained for bare graphene. The low level of ion blocking, in this case, was also related to the formation of conglomerates and aggregation of GO on the surface of the graphene membrane. The low ability to cover defects in the graphene layer resulted in a very low water flow due to there being practically no osmotic pressure.

All prepared membranes showed the stability of operation in more than one osmotic test, moreover, most often the second measurement resulted in a higher water flow and slightly higher ion transport than during the first measurement. It may be related to the intercalation of water molecules between the flakes of reduced graphene oxide and between graphene and the sealing rGO on the border of graphene defects.

**Figure 6:**
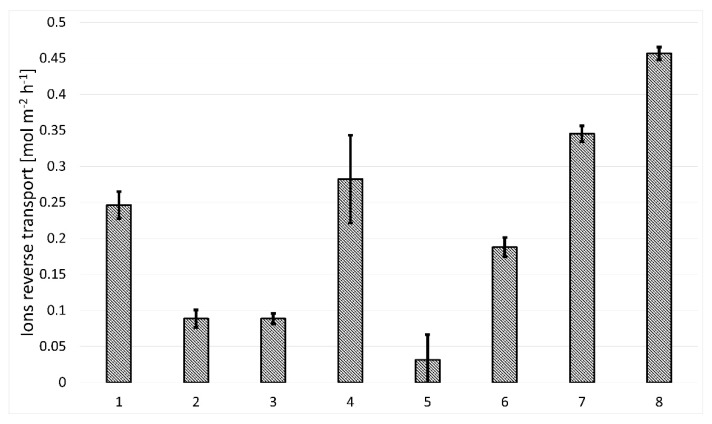
Ion reverse transport through the membrane.

Comparing the test results with the performance of graphene membranes described in the literature, the tested graphene membranes sealed with graphene oxide/reduced graphene oxide show water flow one order of magnitude lower than the water flow of commercially available FO membranes and 2–3 orders of magnitude lower than that obtained by Surwade et al. [8], Jang et al. who used monolayer nanoporous graphene with pores created by ion bombardment and plasma etching [37], as well as monolayer graphene sealed with nylon and hafnia [7]. The ion transport obtained for the membranes presented in this work of 0.3 mol m^−2^ h^−1^ (Figure 6) is of the same order of magnitude as for membranes based on graphene oxide, which, however, exceed the membranes presented in this article by an order of magnitude of water flow [16,38,39,40].

## 4. Conclusions

The study showed that the graphene membranes created are semi-permeable to aqueous solutions. The effectiveness of blocking NaCl ions and the rate of water flow through the membrane are dependent on the degree of GO reduction used to cover HSMG^®^ graphene defects. The degree of GO reduction has been shown to vary depending on the reagent used for this purpose. The best ion blocking level was achieved for graphene membranes covered with reduced graphene oxide. The higher the GO reduction efficiency, the better the ion blocking level. The best solution in this respect proved to be the use of hydrazine and NaBH_4_, with an ion transport blocking efficiency of approximately 90%. However, the high ion blocking efficiency is also associated with low water flow through such membranes. The blocking of the flow of water molecules is most likely due to the reduction of interplanar distances in the rGO. The heavily reduced graphene oxide also loses the ability to selectively cover graphene membranes in places of defects. The even rGO coverage of the HSMG^®^ graphene surface blocks the flow of water molecules through the desired subnanometric defects. The highest water flow was obtained with GO-based membranes. Low or no GO reduction results in the ability to selectively cover HSMG^®^ graphene defects and limit the blocking of water molecules. The highest water flow rate was obtained for membranes made with vitamin C (20 mL m^−2^ h^−1^ bar^−1^). Only the solution based on green tea did not give positive results. Here, it is most likely that the low degree of sealing affected the generation of low osmotic pressure, which translates into low water flow. The optimal solution, from the point of view of the flow rate, and the degree of ion blocking, is the solution using GO reduced with ethylenediamine, with a water flow of 12 mL m^−2^ h^−1^ bar^−1^ and ion blocking at the level of 70%.

## Figures and Tables

**Table 1:** Variant of the graphene oxide reducing agent and the corresponding membrane designation.

Reducing Agent	GO/rGO Sample Number; Membrane Designation
Unreduced graphene oxide	1
Hydrazine (N_2_H_4_) (without centrifugation of reaction products	2
Hydrazine (N_2_H_4_)	3
L(+)-ascorbic acid (C_6_H_8_O_6_)	4
Sodium borohydride (NaBH_4_)	5
Ethylenediamine (C_2_H_8_N_2_)	6
Green tea	7

**Table 2:** Position of Raman spectra peaks for rGO.

Sample No.	G_APP_ Position (cm^−1^)	D’ Position (cm^−1^)	(D’-G_app_)	R^2^
1	1601.10	1600.58	−0.52	0.991
2	1601.56	1614.86	13.30	0.982
3	1600.84	1614.30	13.46	0.988
4	1600.85	1605.86	5.01	0.991
5	1600.85	1608.11	7.26	0.985
6	1603.89	1605.68	1.79	0.990
7	1600.85	1601.35	0.5	0.982

**Table 3:** Structural parameters of reduced graphene oxides resulting from XRD patterns.

Sample No.	Reflection	2theta	FWHM [2Th]	d [A]	D [A]	n
1	(001) GO	16.51	2.37	7.97	25.7	4
2	(001) GO	14.08	0.35	9.34	374	41
	(002)rGO	37	9.8	3.61	13.3	4–5
	(100)GO	43.06	0.4	3.12	327	105
3	(001) GO	---	----	---	---	---
	(002)rGO	33.7	8	3.95	9.5	3
4	(001) GO	18.48	4.8	7.13	11.5	2–3
	(002)rGO	33.9	5	3.92	14.1	4
5	(001) GO	15.96	2.53	8.25	34.2	5
	(002)rGO	33.8	13	3.94	6.8	2–3
6	(001) GO	16.12	1.24	8.16	46.8	7
7	(001) GO	18.93	5	6.96	11.4	2–3

**Table 4:** SEM images of graphene/rGO membranes.

No.	SE Mode	AEE Mode
1	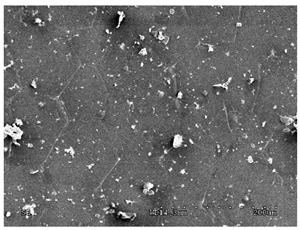	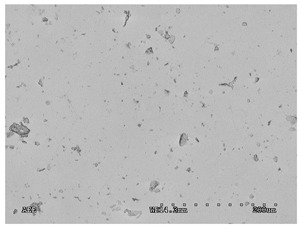
2	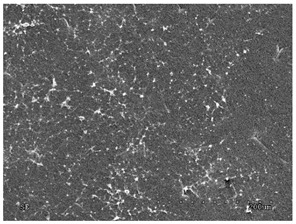	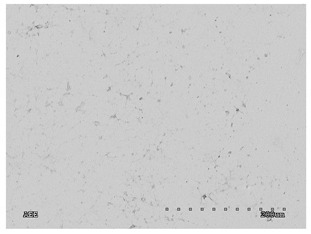
3	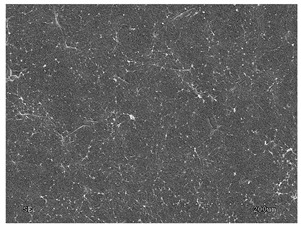	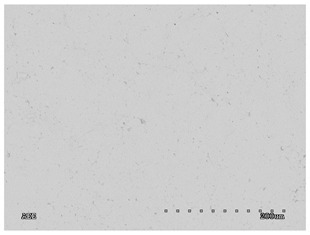
4	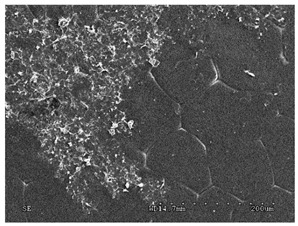	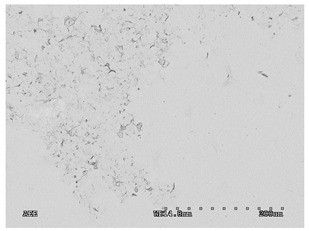
5	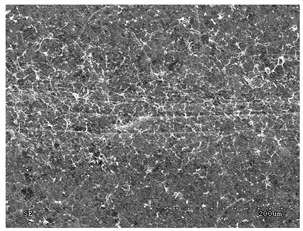	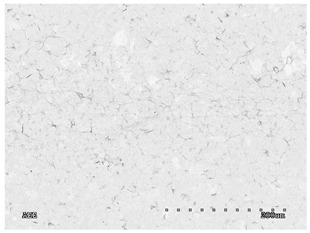
6	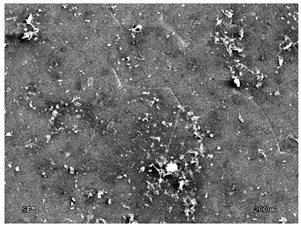	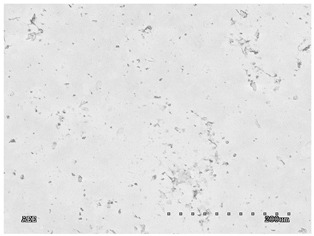
7	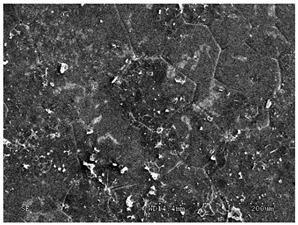	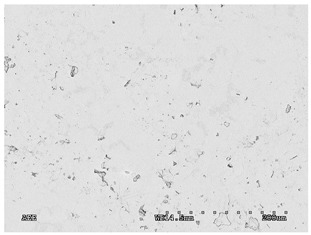
8	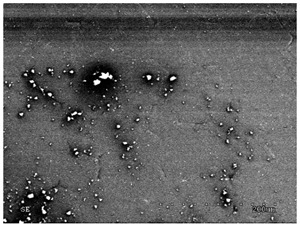	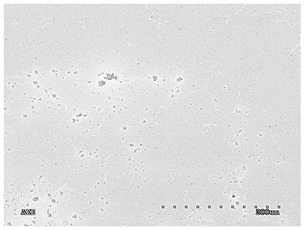

## Data Availability

Not applicable.
